# Single-shot ablation for AF: Real-world procedural and one-year outcomes with the newly adopted PulseSelect PFA compared with cryoballoon from the 1STOP project

**DOI:** 10.1007/s10840-026-02392-w

**Published:** 2026-07-04

**Authors:** Giuseppe Boriani, Giulio Molon, Stefano Nardi, Gianfranco Mitacchione, Danilo Ricciardi, Enrico Chieffo, Roberto Mantovan, Luca Bontempi, Cristian Martignani, Michela Casella, Edoardo Casali, Vincenzo Turco, Alessandro Costa, Luigi Argenziano, Matteo Ziacchi, Antonio Dello Russo

**Affiliations:** 1 Cardiology Division, Department of Biomedical, Metabolic and Neural Sciences, University of Modena and Reggio Emilia, Policlinico di Modena, Policlinico di Modena, Modena, Italy; 2 IRCCS Sacro Cuore Don Calabria, Negrar di Valpolicella, Valpolicella, Italy; 3 Pineta Grande Hospital, Castel Volturno, Italy; 4 Department of Electrophysiology and Cardiac Pacing, Department of Medical and Surgical Specialties, Radiological Sciences and Public Health, ASST Spedali Civili, University of Brescia, Brescia, Italy; 5 Fondazione Policlinico Campus Biomedico, Roma, Italy; 6 Ospedale Maggiore, Crema, Italy; 7 ULSS 2 Marca Trevigiana, Ospedale di Conegliano, Conegliano, Italy; 8 Ospedale Bolognini, Seriate, Italy; 9 Azienda Ospedaliero, Universitaria di Bologna AOUBO IRCCS Policlinico di Sant Orsola, Bologna , Italy; 10 Department of Clinical Sciences, Marche Polytechnic University, Ancona, Italy; 11 Maria Cecilia Hospital, GVM Care & Research, Cotignola, Italy; 12 Department of Biomedical Sciences and Public Health, Marche Polytechnic University, Ancona, Italy

**Keywords:** Atrial Fibrillation, Pulmonary Vein Isolation, Catheter Ablation, Cryoablation, Pulsed Field Ablation, Paroxysmal Atrial Fibrillation, Persistent Atrial Fibrillation

## Abstract

**Background:**

Pulmonary vein isolation (PVI) is an established treatment for symptomatic atrial fibrillation (AF). To date, cryoballoon (CB) ablation has been the most widely adopted single-shot approach, while pulsed field ablation (PFA) has emerged as a non-thermal alternative designed to preferentially ablate myocardial tissue and reduce collateral injury. PulseSelect™ is a CE-marked and FDA approved PFA system specifically developed for PVI. We compared acute and one-year outcomes of PulseSelect PFA versus fourth-generation cryoballoon (CB4) ablation in routine clinical practice.

**Methods:**

This multicenter analysis included 254 consecutive patients undergoing first-time PVI in 10 Italian medium-volume centers between January 2024 and February 2025: 121 treated with PulseSelect and 133 with CB4. Baseline clinical and echocardiographic characteristics were prospectively collected. Procedural workflow, anesthesia strategy, procedure duration, fluoroscopy time, and periprocedural complications were assessed. Follow-up consisted of scheduled visits with rhythm monitoring, and arrhythmia recurrence was evaluated using Kaplan–Meier analysis.

**Results:**

A total of 254 consecutive patients (mean age 62 ± 9 years, 28.3% women with paroxysmal AF in 76.4%) undergoing first-time PVI were included in the analysis: 133 treated with CB4 and 121 with PS PFA, with largely comparable baseline characteristics. General anesthesia was more frequently used with PulseSelect than with CB4 (74.4% vs. 16.5%). Mean skin-to-skin procedure duration was shorter with PulseSelect (62.4 ± 18 min) compared with CB4 (71.4 ± 30 min; *p* = 0.005), while fluoroscopy time was longer with PulseSelect. No acute complications occurred in the PulseSelect group; three transient phrenic nerve palsies were observed with CB4. At 12 months, no statistically significant difference in arrhythmia recurrence was observed between PulseSelect and CB4 (17.4% vs. 15.0%; *p* = 0.46).

**Conclusions:**

In this real-world multicenter cohort, PulseSelect PFA and CB4 ablation showed excellent acute safety and no statistically significant differences in one-year arrhythmia recurrence were observed between the two treatment strategies.

**Supplementary Information:**

The online version contains supplementary material available at https://doi.org/10.1007/s10840-026-02392-w.

## Introduction

Catheter ablation has become an established and effective therapy for patients with symptomatic atrial fibrillation (AF), with pulmonary vein isolation (PVI) representing the cornerstone of contemporary ablation strategies [1–3]. Over the last decade, the adoption of single-shot technologies has substantially simplified procedural workflows, reducing procedure times and standardizing lesion delivery compared with conventional point-by-point radiofrequency ablation [2, 4]. Among these approaches, cryoballoon ablation has emerged as the most widely adopted single-shot technique and is a reference standard in many electrophysiology laboratories worldwide [4–12].

More recently, pulsed field ablation (PFA) has been introduced as a novel, non-thermal energy source based on irreversible electroporation, enabling preferential myocardial tissue ablation while minimizing injury to surrounding structures such as the esophagus, phrenic nerve, and pulmonary veins. Early clinical experience with PFA has demonstrated encouraging safety and efficacy profiles, fostering rapid interest and adoption in routine clinical practice [13–18]. The PulseSelect™ system (Medtronic Inc, Minneapolis) is a CE-marked PFA technology specifically designed for single-shot pulmonary vein isolation [19, 20]. The system combines a circular multielectrode catheter with bipolar, biphasic pulsed-field energy delivery, aiming to achieve rapid and reproducible lesion formation with simplified procedural workflows. While early feasibility and registry data have supported the safety of PulseSelect PFA (PS PFA), comparative real-world evidence against established single-shot technologies remains limited. Certainly, there are few real-world data collections whereby the two systems (PulseSelect and Cryo) have been compared within the same registry framework with similar/same operators from the same centers using similar/same standard operation procedures.

Within the framework of the One Shot to PVI (1STOP) project, we sought to compare acute procedural characteristics, safety, and one-year clinical outcomes of PS PFA with those of the fourth-generation cryoballoon ablation (CB4), as performed in routine clinical practice across medium-volume Italian centers. This analysis reflects the early adoption phase of PS PFA and contrasts the data with the most recent experience using cryoballoon technology, providing insight into workflow differences and clinical outcomes in a contemporary real-world setting.

## Methods

### Study design and patient population

This was a prospective, multicenter, observational cohort analysis conducted within the One Shot to PVI (1STOP) project. Consecutive patients undergoing first-time PVI for symptomatic atrial fibrillation were included. Procedures were performed between January 2024 and February 2025 across 10 Italian centers routinely performing both pulsed field and cryoballoon ablation. Patients were eligible if they had symptomatic paroxysmal or persistent AF and were undergoing *de novo* catheter ablation with a single-shot technique. Patients with prior left atrial ablation, surgical AF ablation were excluded. The study population comprised two contemporaneous groups defined by the ablation technology used (i) patients treated with the PulseSelect™ pulsed field ablation system, representing the early clinical adoption phase of PFA at participating centers and (ii) patients treated with a fourth-generation 28-mm cryoballoon (Arctic Front Advance Pro™, Medtronic), representing the most recent cryoballoon experience at the same centers. The comparison reflects real-world consecutive practice, contrasting the initial PulseSelect experience with the latest cryoballoon procedures.

### 1STOP project framework and ethics

The 1STOP project is part of the broader One Hospital ClinicalService initiative, a medical care quality improvement program designed to collect real-world data on Medtronic ablation technologies with the aim of improving procedural quality and clinical outcomes. Data were prospectively collected using a centralized electronic database. All procedures and data collection were conducted in accordance with the Declaration of Helsinki. The One Hospital ClinicalService program was approved according to local regulatory requirements at each participating center, and all patients provided written informed consent for inclusion and follow-up.

### Baseline data collection

Baseline clinical, demographic, and echocardiographic characteristics were collected for all patients, including age, sex, body mass index, AF type and duration, cardiovascular comorbidities, left atrial size, left ventricular ejection fraction, and ongoing pharmacological therapy at the time of ablation.

### Procedural characteristics

All procedures were performed according to standard clinical practice at each center. The choice of ablation technology was at the discretion of the treating physician and center availability. Procedural data collected included, but were not limited to (i) type of anesthesia (general anesthesia or conscious sedation); (ii) total procedure duration (skin-to-skin); (iii) fluoroscopy time; (iv) left atrial dwell time, defined as the interval from transseptal puncture to final catheter removal from the left atrium; (v) cumulative number of pulsed-field applications or cryothermal freezes per PV; and (vi) acute procedural success, defined as documented entrance or exit block of all pulmonary veins. Acute pulmonary vein isolation was assessed according to local practice. In most patients, isolation was confirmed by pacing maneuvers demonstrating conduction block. When electroanatomical mapping systems were used, additional confirmation was obtained through voltage and/or activation mapping. Routine three-dimensional remapping after ablation was performed at the discretion of the operator. Periprocedural complications were recorded and defined as any adverse event occurring from the start of the procedure until hospital discharge.

### Follow-up and clinical outcomes

After ablation, patients were followed according to each center’s standard clinical practice, including scheduled outpatient visits and rhythm monitoring. A 24-hour Holter recording was performed at 12 months after the index procedure. Outcome analyses were performed applying both 90-day and 60-day blanking periods; additional analyses were conducted without the application of a blanking period. The primary clinical endpoint was arrhythmia recurrence at 12 months, defined as any documented episode of atrial fibrillation, atrial tachycardia (AT) or atrial flutter (AFL) lasting ≥ 30 s beyond the blanking period. Time-to-event analyses were performed using Kaplan–Meier estimates.

### Statistical analysis

Descriptive statistics were used to summarize all results. These include mean and standard deviation, minimum, maximum and median with interquartile range (IQR) for continuous variables and counts and percentages for categorical variables. Summary statistics are reported with maximum 2 decimals, as appropriate. Continuous variables were compared between groups using Wilcoxon’s rank sum test. Categorical variables were compared between groups using Chi-square test or Fisher’s exact test, as appropriate. Variables may also be represented graphically, using boxplots and bar charts for continuous and categorical variables, respectively, whenever appropriate. Occurrence of AF recurrence was analyzed as time-to-first event using Kaplan-Meier curves (with the corresponding 95% confidence intervals, 95%CIs), compared with log-rank test. Multivariable Cox proportional hazards regression analyses were performed to assess the association between ablation technology and arrhythmia recurrence. Given the limited number of recurrence events observed during follow-up, the number of covariates included in each model was restricted to avoid overfitting. Two separate models were therefore constructed, including clinically relevant variables such as age, AF type, and anesthesia strategy. All statistical tests are based on a two-sided significance level of 0.05. SAS software, version 9.4, (SAS Institute Inc., Cary, NC, USA)

## Results

### Patient population

A total of 254 consecutive patients undergoing first-time PVI were included in the analysis: 133 treated with CB4 and 121 with PS PFA. Overall, the study population had a mean age of 61.9 ± 9.1 years, and 28.3% were women. Baseline demographic characteristics, including age distribution, sex, body mass index, and cardiovascular risk profile, were well balanced between the two groups (Table 1). Paroxysmal atrial fibrillation was the predominant arrhythmia type, accounting for 76.4% of cases, with no significant differences between the CB4 and PS PFA cohorts. The prevalence of persistent or long-standing persistent AF was similarly low and comparable across groups. With respect to comorbidities, hypertension was highly prevalent and similar between groups. Stroke and transient ischemic attack were infrequent and evenly distributed. Thromboembolic risk, assessed by CHA₂DS₂-VASc was low overall and comparable between treatment groups.

**Table 1:** Baseline characteristics of patients undergoing cryoballoon ablation or PulseSelect pulsed field ablation

Baseline characteristics	Total (*N* = 254)	Cryoablation CB4 (*N* = 133)	PulseSelect PS PFA (*N* = 121)	*p*-value
Gender (Female)	28.3% (72/254)	26.3% (35/133)	30.6% (37/121)	0.487
Age at first ablation (years)	61.9 ± 9.1	61.6 ± 9.4	62.2 ± 8.8	0.571
Body Mass Index (Kg/m^2^)	26.7 ± 4.2	26.6 ± 4.3	26.8 ± 4.0	0.735
Type of Atrial Fibrillation				0.304
Paroxysmal	76.4% (194/254)	73.7% (98/133)	79.3% (96/121)	
Persistent / Long Standing Persistent	23.6% (60/254)	26.3% (35/133)	20.7% (25/121)	
Median of Months from AF diagnosis to PVI	24.0 (12.0–60.0)	24.0 (12.0–60.0)	19.0 (10.0–50.0)	0.184
First Line	13.8% (35/254)	17.3% (23/133)	9.9% (12/121)	0.104
Previous tested AADs ≥ 1	86.2% (219/254)	82.7% (110/133)	90.1% (109/121)	0.104
Median EHRA score	2.0 (2.0–2.0)	2.0 (1.0–2.0)	2.0 (2.0–2.0)	0.308
History of Stroke/TIA	3.1% (8/254)	3.0% (4/133)	3.3% (4/121)	1.000
Heart failure				0.130
NYHA < 2	80.3% (204/254)	76.7% (102/133)	84.3% (102/121)	
NYHA > = 2	19.7% (50/254)	23.3% (31/133)	15.7% (19/121)	
Hypertension	52.0% (132/254)	52.6% (70/133)	51.2% (62/121)	0.900
Any Valve Disease	16.7% (41/246)	14.4% (18/125)	19.0% (23/121)	0.393
Mean CHA₂DS₂-VASc	1.6 ± 1.3	1.6 ± 1.3	1.5 ± 1.3	0.878
Diabetes	7.1% (18/254)	5.3% (7/133)	9.1% (11/121)	0.328
Chronic Kidney Disease	2.9% (7/238)	3.4% (4/117)	2.5% (3/121)	0.719
Left Ventricular EF %	57.6 ± 6.1	56.9 ± 6.7	58.3 ± 5.4	0.097
Indexed LA Diameter* (median I-III IQR) mm/mm^2^	26.0 (20.2–27.0)	18.8 (17.5–20.2)	26.5 (23.6–27.7)	0.107
Class I or III AADs	61.8% (157/254)	60.2% (80/133)	63.6% (77/121)	0.606

Abbreviations: *AF* atrial fibrillation, *AADs* antiarrhythmic drugs, *PVI* Pulmonary Vein Isolation, *TIA* transient ischemic attack, *LVEF* left Ventricular Ejection Fraction, *LA* left atrium, *CV* Cardiovascular

### Procedural findings and complications

Procedural characteristics are summarized in Table 2. A marked difference emerged in anesthesia strategy between groups: general anesthesia was used in most of PS PFA procedures (74.4%), whereas it was adopted in a minority of cryoballoon cases (16.5%), reflecting different approaches, workflow organization and room occupancy requirements.

**Table 2:** Procedural data according to PVI technology

Procedural Characteristics	Total(N = 254)	Cryoablation CB4(N = 133)		PulseSelect PS PFA(N = 121)	p-value
Type of anesthesia performed					<0.001
General anesthesia (initial choice)	47.2% (120)	16.5% (22)		74.4% (90)	
Moderate/deep sedo-analgesia (initial choice)	52.0% (132)	83.5% (111)		24.0% (29)	
Moderate/deep sedo-analgesia (upgrade from mild during the procedure)	0.8% (2)	0.0% (0)		1.7% (2)	
Procedural Measurements					
Procedure Duration (min)	67.1 ± 25.9	71.4 ± 30.8		62.4 ± 18.2	0.005
Fluoroscopy Duration (min)	18.1 ± 10.1	16.1 ± 11.4		20.2 ± 8.3	0.002
Acute success Rate	98.9% (251)	97.7% (130)		100%% (121)	0.427
Method used to verify left atrial access					
TEE	40.2% (102)	34.6% (46)		46.3% (56)	0.073
ICE	16.5% (42)	9.8% (13)		24.0% (29)	0.004
Fluoroscopy	42.5% (108)	36.8% (49)		48.8% (59)	0.058
Guie	10.6% (27)	10.5% (14)		10.7% (13)	1.000
No Mapping	84.6% (215)	81.4% (108)		87.8% (106)	0.005
Pre-ablation rhythm					0.782
Sinus	75.0% (189)	73.5% (97)		76.7% (92)	
Atrial Fibrillation	23.8% (60)	25.0% (33)		22.5% (27)	
Atrial Flutter	1.2% (3)	1.5% (2)		0.8% (1)	
Post-ablation rhythm					0.474
Sinus	93.0% (214/230)	93.1% (108/116)		93.0% (106/114)	
Atrial Fibrillation	6.1% (14/230)	6.9% (8/116)		5.3% (6/114)	
Atrial Flutter	0.9% (2/230)	0.0% (0/116)		1.8% (2/114)	
Left Superior Pulmonary Vein					
No. Freezes cryo	-		1.3 ± 0.5	-	-
No.Applications PFA	-		-	9.3 ± 1.8	-
Left Inferior Pulmonary Vein					
No. Freezes cryo§	-		1.3 ± 0.6		-
No.Applications PFA§	-			8.5 ± 1.5	-
Right Superior Pulmonary Vein					
No. Freezes cryo §	-		1.3 ± 0.6	-	-
No.Applications PFA §	-			8.5 ± 1.2	-
Phrenic nerve monitored	97.6% (248/254)		98.9% (132/133)	96.6% (117/121)	0.388
Right Inferior Pulmonary Vein					
No. Freezes cryo§	-		1.3 ± 0.5	-	-
No.Applications PFA §	-		-	8.5 ± 1.6	-
Phrenic nerve monitored	97.6% (248)		100.0% (133)	95.7% (116)	0.067
Left common ostium	7.9% (20)		10.5% (14)	5.0% (6)	0.109
No. Freezes cryo §	-		1.7 ± 0.9	-	-
No.Applications PFA§	-		-	10.5 ± 3.9	-
Accessory Pulmonary Vein	1.2% (3)		1.5% (2)	0.8% (1)	1.000
No. Freezes cryo§	-		1.0 ± 0.0	-	-
No.Applications PFA §	-			8.0 ± NA	-
Left Atrial Additional lines	7.5% (19)		6.0% (8)	9.1% (11)	0.465

The number of applications/freezes is technology-specific and not directly comparable; therefore, no statistical comparison was performed

Data are presented as mean+/-Standard deviation for continuous variables or % (n) for categorical variables. *AV* Arterio-Venous, *PVI* pulmonary vein isolation, *TEE* Transesophageal Echocardiograph, *ICE*  Intracardiac echocardiography.Guide: advancement of a guidewire across the interatrial septum through the transseptal system  to provide fluoroscopic confirmation of successful left atrial access.

Total procedure duration (skin-to-skin) was significantly shorter in the PulseSelect cohort (62.4 ± 18 min) compared with cryoballoon ablation (71.4 ± 30 min; *p* = 0.005). Fluoroscopy time was modestly longer with PulseSelect (20.2 ± 8 vs. 16.1 ± 11 min; *p* = 0.002). A three-dimensional mapping system was employed in a minority of cases (overall 15.4%), without significant between-group differences (cryoablation 18.6% vs. PulseSelect 12.2%, *p* = 0.202). An additional analysis was performed within the PulseSelect cohort according to the use of electroanatomical mapping systems. Procedures performed with mapping were associated with longer procedure duration compared with procedures performed without mapping (72.6 ± 17.1 vs. 61.4 ± 18.3 min, *p* = 0.010). However, fluoroscopy duration was similar between groups (18.7 ± 8.0 vs. 20.6 ± 8.4 min, *p* = 0.468).

Among the 254 patients, 60 (23.8%) were in atrial fibrillation at the time of the procedure. Overall, 49 of these patients (81.7%) converted to sinus rhythm during the procedure. Conversion rates were similar between treatment groups, occurring in 28 of 33 patients (84.8%) undergoing cryoballoon ablation and in 21 of 27 patients (77.8%) treated with PulseSelect PFA (*p* = 0.520).

Acute procedural success, defined as complete PVI, was achieved in nearly all treated veins, with comparable acute isolation rates across groups (97.7% in CB4 group vs. 100% in PS PFA group, *p* = 0.426). Acute pulmonary vein isolation was most commonly documented by pacing maneuvers (75.0%), whereas voltage mapping and activation mapping were used in 8.8% and 3.8% of patients, respectively. Additional left atrial ablation lines beyond PVI were infrequently performed (7.5%) and did not differ significantly between cohorts (cryoablation 6.0% vs. PulseSelect 9.1%, *p* = 0.475). Post-procedural atrial flutter occurred in two patients, both treated with PulseSelect PFA. Both cases were successfully managed with antiarrhythmic drug therapy, without the need for electrical cardioversion or repeat ablation.

Periprocedural complications were uncommon. No acute complications were observed in the PulseSelect group and particularly no cases of coronary spasm were observed. In the cryoballoon cohort, transient phrenic nerve palsy was observed in three patients (all resolving, two early and one within three months). There were no phrenic nerve injuries with the usage of PulseSelect. Table 3.

**Table 3:** Acute procedural complications in the overall study population and according to treatment group (Cryoballoon ablation vs. PulseSelect pulsed field ablation)

Procedural characteristics	Total (*N* = 254)	Cryoablation CB4 (*N* = 133)	PulseSelect PS PFA (*N* = 121)	*p*-value
Patients with ≥ 1 complication	1.2% (3)	2.2% (3)	0.0% (0)	1.000
Transient Diaphragmatic Paralysis	1.2% (3)	2.2% (3)	0.0% (0)	1.000
Peri-cardial Effusion	0.0% (0)	0.0% (0)	0.0% (0)	N/A
AV Fistula	0.0% (0)	0.0% (0)	0.0% (0)	N/A
Cardiac Tamponade	0.2% (0)	0.3% (0)	0.0% (0)	N/A
Pneumothorax/Hemothorax	0.0% (0)	0.0% (0)	0.0% (0)	N/A
Femoral Pseudo-aneurism	0.0% (0)	0.0% (0)	0.0% (0)	N/A
Other Minor Complications	0.0% (0)	0.0% (0)	0.0% (0)	N/A

### One-year clinical outcomes

**Fig. 1:**
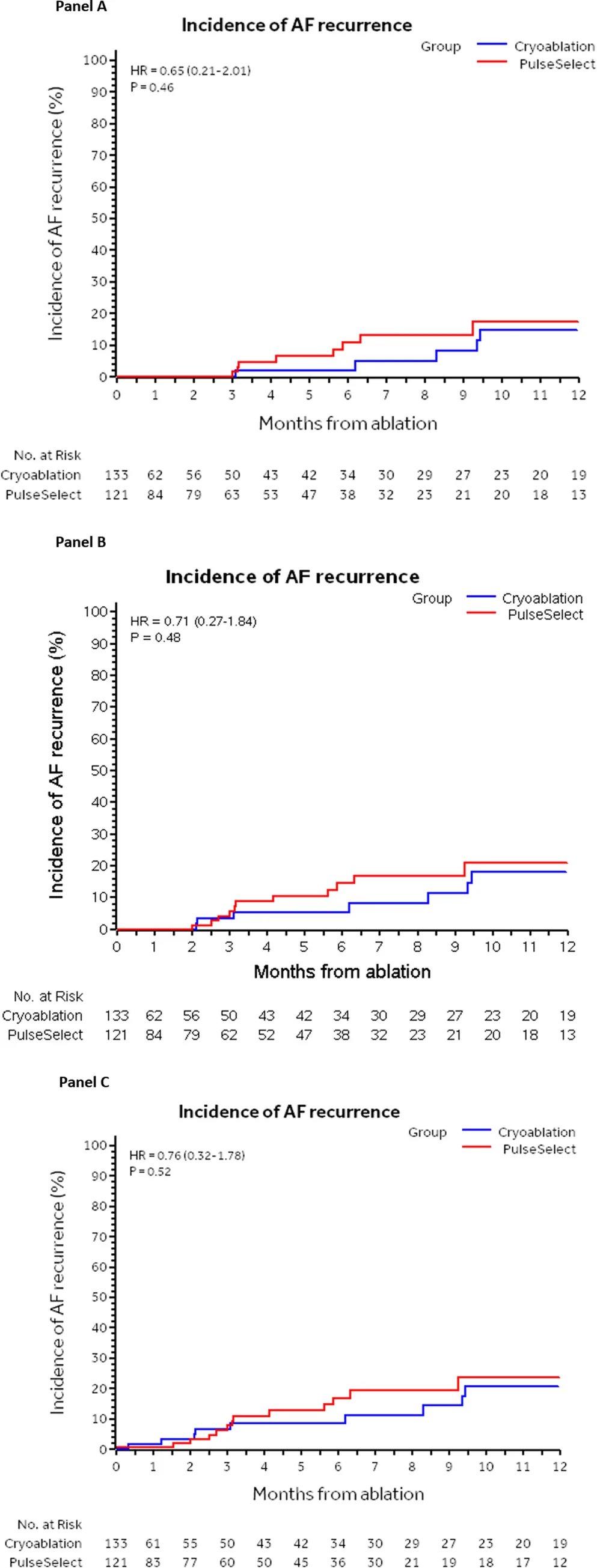
Kaplan–Meier estimates of cumulative incidence of AF recurrence after pulmonary vein isolation with fourth-generation cryoballoon (CB4) versus PulseSelect pulsed field ablation (PFA). Panel **A**: 90-day blanking period. Panel **B**: 60-day blanking period. Panel **C**: no blanking period. No significant differences in event incidence were observed between ablation strategies across analyses

At 1-year follow-up, arrhythmia-free survival was comparable between cryoballoon ablation and PulseSelect pulsed field ablation across all analytical approaches, including different blanking period definitions (Figures panel A-C). Atrial arrhythmia recurrence was defined as AF, AT, or AFL > 30 s. All observed recurrences consisted of AF episodes. Analysis with a 90-day blanking period showed a cumulative incidence of atrial arrhythmia recurrence at 12 months of 15.0% (95% CI: 6.4–32.8%) in the CB and 17.4% (95% CI: 8.6–33.4%) in the PulseSelect group (Fig. 1), with no significant between-group difference and overlapping confidence intervals throughout follow-up.

**Fig. 2:**
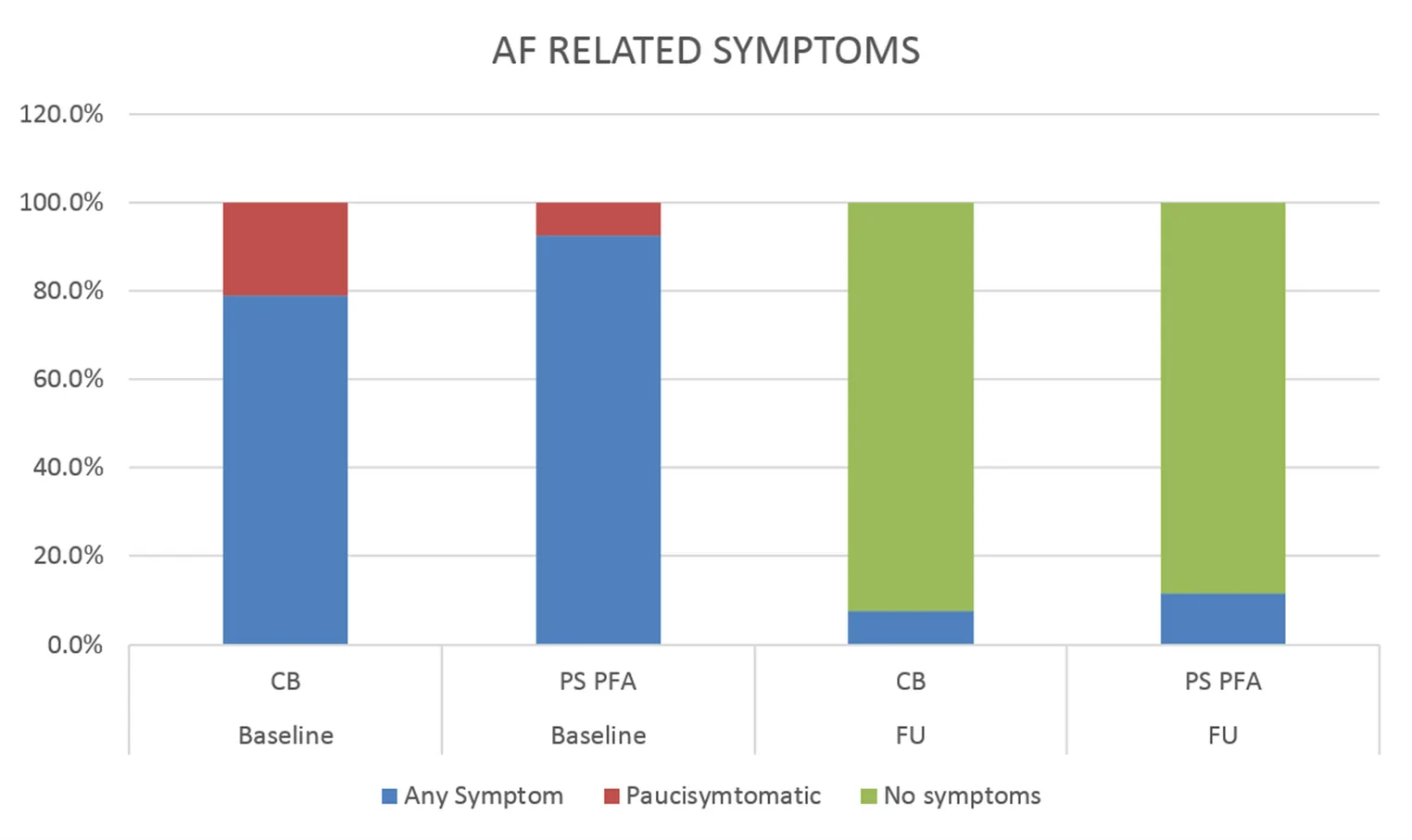
Change in symptom burden from baseline to last follow-up. At baseline, the majority of patients reported atrial fibrillation–related symptoms, with a smaller proportion being paucisymptomatic. At last follow-up, only a minority of patients reported recurrent symptoms, while most patients were asymptomatic, with no significant differences between ablation technologies

When a 60-day blanking period was applied, recurrence rates at one year were slightly higher but remained comparable between groups, with cumulative incidences of 18.0% (95% CI: 8.7–35.0%) in the cryoballoon cohort and 20.9% (95% CI: 11.5–36.2%) in the PulseSelect cohort (Fig. 2). Time-to-event analysis confirmed no significant difference in arrhythmia recurrence between ablation strategies (hazard ratio 0.71, 95% CI: 0.27–1.84). In analyses performed without applying any blanking period, early post-procedural arrhythmia episodes contributed to higher absolute event rates in both groups.

To further explore the potential impact of confounding factors, multivariable Cox proportional hazards regression analyses were performed. Given the limited number of recurrence events observed during follow-up, two separate models were constructed to avoid overfitting. In Model 1, including ablation technology, anesthesia strategy, and AF type, ablation technology was not independently associated with arrhythmia recurrence (HR 2.305, 95% CI 0.772–6.882; *p* = 0.135). Similarly, in Model 2, including ablation technology, age, and AF type, no significant association was observed between ablation technology and recurrence (HR 1.438, 95% CI 0.550–3.762; *p* = 0.459). Detailed results are provided in Supplementary Tables S1A and S1B.

A sensitivity analysis was performed including patients who only underwent standalone pulmonary vein isolation, excluding those treated with additional left atrial ablation lines and those requiring ablation beyond the four standard pulmonary veins. The analysis included 217 patients (112 cryoballoon and 105 PulseSelect). AF recurrence at 12 months occurred in 5.4% of patients treated with cryoballoon ablation and 10.5% of those treated with PulseSelect PFA, with no statistically significant difference between groups (*p* = 0.208).

At baseline, in accordance with established indications for catheter ablation, all patients were symptomatic. AF-related symptoms were reported by 78.9% of patients in the cryoballoon group and by 92.6% in the PulseSelect PFA group. The remaining patients were paucisymptomatic with EHRA score equal or less than 2 (21.1% and 7.4%, respectively). At follow-up, a marked improvement in symptom burden was observed in both treatment groups (Fig. 2). Residual symptoms were uncommon, with any AF-related symptom reported by only 9.4% of the overall population, with comparable rates between cryoballoon and PulseSelect PFA (7.5% vs. 11.6%, respectively; *p* = 0.291). (Fig. 4) Among patients reporting residual symptoms, palpitations were the most frequently described complaint (83.3%), whereas dyspnea (4.2%), asthenia (8.3%), and other symptoms (4.2%) were rare and evenly distributed between ablation strategies During the first 12 months of follow-up, only one repeat ablation procedure was performed, occurring in a patient treated with cryoballoon ablation who experienced symptomatic AF recurrence.

### Learning curve analysis

Based on the previously published 1STOP PulseSelect learning-curve analysis by Molon et al. [21], patients were categorized according to center experience as novice (≤ 2 procedures) or experienced (> 2 procedures). Procedure duration was significantly longer during the initial adoption phase (72.9 ± 14.2 vs. 60.6 ± 18.2 min, *p* < 0.001), whereas fluoroscopy time was comparable between experience groups (21.4 ± 8.7 vs. 20.0 ± 8.3 min, respectively; *p* = 0.219). AF recurrence was numerically higher among novice operators (16.7% vs. 7.8%), although the difference did not reach statistical significance (*p* = 0.210).

## Discussion

This multicenter, real-world analysis from the 1STOP project describes one of the first multicentric comparative evaluations of PFA using the PS PFA system versus CB4 in patients undergoing first-time AF ablation. The main findings can be summarized as follows: (i) both technologies achieved high acute success rates with a very low incidence of complications; (ii) one-year arrhythmia-free survival did not differ significantly between groups across all analytical approaches, including different blanking period definitions; (iii) a marked and consistent improvement in AF-related symptoms was observed irrespective of the ablation technology used; however, (iv) there were differences in the procedures between the two catheter systems with PulseSelect procedures implying a more frequent use of general anesthesia and cryoballoon having more phrenic nerve injury.

### Ease of adoption

In the participating centers, who had extensive experience in single-shot pulmonary vein isolation using cryoballoon technology, the transition to PS PFA proved to be straightforward and did not compromise procedural performance or clinical outcomes. In our cohort, PS PFA was introduced during the earliest phase of adoption in each participating center, yet acute isolation rates and one-year rhythm outcomes were comparable to those achieved with a mature cryoballoon program. This observation is consistent with the multicenter experience reported by Molon et al., who described a very short learning curve, limited to approximately 2–3 cases per center, during the initial clinical use of PS PFA across six Italian sites, most of which were high-volume cryoballoon centers [21]. To further explore the impact of operator experience in the present cohort, we performed an additional analysis using the same threshold identified by Molon et al. (≤ 2 versus > 2 procedures per center). Procedure duration was significantly longer during the earliest adoption phase (72.9 ± 14.2 vs. 60.6 ± 18.2 min, *p* < 0.001), confirming a rapid gain in procedural efficiency. In contrast, fluoroscopy duration remained similar between groups (21.4 ± 8.7 vs. 20.0 ± 8.3 min, *p* = 0.219). Although AF recurrence was numerically higher in the earliest cases (16.7% vs. 7.8%), this difference did not reach statistical significance (*p* = 0.210), suggesting that the learning curve primarily affected procedural efficiency rather than clinical effectiveness. When placed in the broader context of PFA literature, these findings are further supported by large real-world PFA registries [22–26]. Similar to our experience, FRANCE-PFA highlighted the ease of implementation of PFA in routine clinical workflows and the rapid operator confidence gained with the technology [26]. The US large Disrupt-AF registry showed a rapid physician learning curve by substantial reductions in procedural and fluoroscopy times as operator experience increased (> 11 procedures) while maintaining a favorable safety profile [22].

### Procedural data

In our analysis, procedural duration was significantly shorter in the PulseSelect group compared to cryoballoon (mean 62.4 vs. 71.4 min; *p* < 0.001). However, this metric must be interpreted in light of the anesthetic approach: approximately 80% of cryoablations were performed under moderate sedation, while the majority of PulseSelect procedures required general anesthesia. This distinction has important implications. While cut-to-cut procedure times are shorter with PFA, general anesthesia typically prolongs total lab workflow due to the additional time required for induction and recovery. Prior observational studies have shown that general anesthesia may extend overall EP lab use by approximately 30–35 min compared to conscious sedation [27–28]. However also deep sedation has been largely applied in selected experiences on PFA [29–31]. Interestingly, fluoroscopy time was modestly longer in the PulseSelect group despite the shorter overall procedure duration. Several factors may explain this finding. First, the present study reflects the early adoption phase of the technology, during which operators may have relied more extensively on fluoroscopic guidance while becoming familiar with catheter handling and positioning. Second, the PulseSelect workflow involves multiple catheter repositioning maneuvers and repeated energy applications within each pulmonary vein, which may contribute to greater fluoroscopy utilization despite shorter overall procedure duration. Importantly, an additional analysis within the PulseSelect cohort demonstrated that fluoroscopy duration was not significantly influenced by the use of electroanatomical mapping systems, suggesting that differences in mapping utilization are unlikely to explain the fluoroscopy findings observed between technologies.

The use of procedural imaging also differed between groups, with intracardiac echocardiography being more frequently employed during PulseSelect procedures. This difference likely reflects local operator preference and center-specific workflows rather than a technology-specific requirement. Although ICE may facilitate transseptal access and catheter manipulation, its impact on long-term rhythm outcomes remains uncertain.

Moreover, procedural duration should also be interpreted in the context of operator experience. Cryoballoon ablation has been routinely performed in these centers for over a decade, well beyond the learning curve, with highly standardized workflows [32]. In contrast, the PulseSelect system has only recently been introduced. The present analysis includes the first consecutively treated patients in each center, during the earliest phase of adoption. This highlights the intrinsic efficiency of technology even in early experience, and future data may show further improvements as operators gain familiarity. Interestingly, approximately 20% of PulseSelect cases in our cohort were performed under deep sedation, suggesting that the feasibility of less invasive anesthetic strategies is worth further investigation. Additional studies are warranted to evaluate the safety, tolerability, and impact on outcomes of deep sedation in PFA procedures [33–35].

Therefore, comparisons of procedural efficiency between technologies should be interpreted in the context of both the markedly different anesthesia strategies and the different stages of technology adoption observed in the present study.

### Safety

The safety profile of both technologies was favorable in the present multicenter experience. No periprocedural complications were observed in the PulseSelect group, whereas three cases of transient phrenic nerve palsy occurred in the cryoballoon group, all patients showing some symptom resolution before hospital discharge. Phrenic nerve injury is a well-recognized complication of cryoballoon ablation and represents one of the most frequently reported cryo-specific adverse events, particularly during ablation of the right superior pulmonary vein. Large, randomized trials and real-world registries have consistently reported transient phrenic nerve palsy rates ranging from approximately 1% to 2.5%, with persistent injury occurring in a smaller but clinically relevant proportion of patients, despite systematic phrenic nerve monitoring and contemporary preventive strategies [5, 36].

In contrast, no acute complications were observed in patients treated with PS PFA. This finding is consistent with available evidence from clinical studies evaluating the safety of this technology. In the PULSED AF pivotal trial, which evaluated PulseSelect PFA for paroxysmal and persistent AF, the primary safety endpoint—defined as the occurrence of serious procedure- or device-related adverse events—was met, with a low major complication rate of 0.7% and no reported cases of atrioesophageal fistula, pulmonary vein stenosis, or persistent phrenic nerve injury [20]. Similarly, earlier feasibility studies of PulseSelect demonstrated a favorable acute safety profile, supporting the reproducibility of these results across different centers and patient populations [19, 21]. Of note, no case of coronary spasm was observed in the PulseSelect group [16–38].

The favorable safety outcomes observed with PulseSelect PFA are mechanistically linked to the non-thermal nature of irreversible electroporation, which preferentially affects myocardial cells while sparing surrounding structures such as the phrenic nerve, esophagus, and pulmonary veins. This tissue selectivity may translate into a lower risk of collateral injury compared with thermal energy sources, a hypothesis increasingly supported by accumulating clinical data. However, the present analysis was not powered to compare safety outcomes between treatment groups, and the low number of observed complications precludes meaningful statistical comparisons. Therefore, although no complications were observed in the PulseSelect group and three transient phrenic nerve palsies occurred in the cryoballoon group, no conclusions regarding differences in safety profiles can be drawn from the present analysis. Larger comparative studies and longer follow-up will be essential to further clarify potential differences in safety outcomes between PFA and established thermal technologies.

### 1-Year outcomes

In this real-world multicenter cohort, no statistically significant differences in one-year arrhythmia-free survival were observed between CB4 ablation and PS PFA across all analytical approaches. Using a standard 90-day blanking period, recurrence rates at 12 months were approximately 15–17% in both groups, with overlapping confidence intervals and no significant differences. Importantly, our cohort reflects routine clinical practice and included a proportion of patients with persistent AF, a population often underrepresented in early ablation trials. These findings are consistent with the contemporary cryoballoon series [12, 39, 40] and align closely with the results of the PULSED AF pivotal trial, which demonstrated favorable 12-month efficacy of PulseSelect PFA in a population including both paroxysmal and persistent AF patients [20].

These findings suggest that early recurrences affected both technologies similarly and did not translate into divergent long-term rhythm outcomes, even if the duration of blanking period is changed [12]. While early atrial arrhythmias have traditionally been attributed to transient post-ablation inflammation, growing evidence indicates that they may also carry prognostic significance for late recurrence; nonetheless, the optimal duration of the blanking period remains debated [41, 42].

Our findings are aligned with recent evidence from the *CHAMPION-AF* randomized trial, which compared PFA (Farapulse™ system) with cryoballoon ablation. The study demonstrated non-inferiority of PFA in terms of arrhythmia recurrence at 12 months, with a trend toward improved outcomes in the PFA arm (freedom from AF recurrence: 63% vs. 49%, *p* < 0.05) [43]. Importantly, differences in reported outcomes between studies should be interpreted in light of the rhythm monitoring strategy employed. CHAMPION-AF utilized continuous rhythm monitoring with implantable devices, providing substantially greater sensitivity for arrhythmia detection than conventional follow-up based on scheduled visits and intermittent Holter recordings. As a result, a higher proportion of asymptomatic recurrences may be identified, potentially leading to higher reported recurrence rates. Therefore, direct comparisons of absolute recurrence rates between studies should be interpreted with caution. Studies evaluating real-world practice inevitably reflects the variable access to technologies, also in term of continuous monitoring that actually characterize European countries, also with markedly different reimbursement practices affecting the possibility [44]. Despite differences in monitoring strategies, energy platforms, catheter design, and follow-up management, the overall consistency of findings across studies strengthens the clinical relevance of our observations.

Beyond rhythm outcomes, a marked and clinically meaningful improvement in symptom burden was observed in both treatment groups. At one-year follow-up, residual AF-related symptoms were uncommon, reported by less than 10% of patients overall, with no significant difference between ablation strategies. Among symptomatic patients, palpitations were the predominant complaint, whereas dyspnea, asthenia, and other symptoms were rare. These findings are consistent with prior studies showing that successful AF ablation translates into substantial symptom relief and quality-of-life improvement, irrespective of the specific ablation technology used [45].

## Limitations

This is a retrospective analysis of a prospectively collected real-world project and is therefore subject to the inherent limitations of observational studies. Because the two technologies were introduced in different time periods, temporal bias cannot be fully excluded: residual confounding related to operator experience and learning curve effects—particularly during early PFA adoption—may have influenced procedural performance and AF recurrence rates. Furthermore, cryoballoon ablation represented a mature and highly standardized technology in participating centers, whereas PulseSelect PFA was evaluated during its initial implementation phase. This imbalance reflects an inherent challenge of real-world comparative studies involving newly introduced technologies, where early comparative evidence must necessarily be generated before completion of the learning curve. Although additional multivariable analyses were performed, residual confounding cannot be completely excluded. The marked imbalance in anesthesia strategy between groups may have influenced procedural workflow and procedural time comparisons. Differences in procedural imaging strategies, including the use of intracardiac echocardiography, may have contributed to procedural heterogeneity between treatment groups. The analysis is further limited by the sample size and follow-up protocol and duration. In line with the real-world design of the 1STOP project, procedural strategies and follow-up protocols were not standardized across participating centers. As a consequence, asymptomatic AF recurrences may have remained undetected, potentially leading to an overestimation of arrhythmia-free survival. Furthermore, since our report delt with real life conditions, continuous rhythm monitoring was not routinely performed, precluding a comprehensive assessment of AF burden over time. Radiation exposure was assessed using fluoroscopy duration only, as dose-related parameters such as Dose Area Product (DAP) and Air Kerma were not systematically collected within the database. Consequently, a comprehensive comparison of radiation burden between treatment groups could not be performed Finally, the analysis focused on time to first AF recurrence and did not account for AF burden or recurrent episodes, which may provide a more comprehensive assessment of treatment efficacy [46].

## Conclusion

In this real-world multicenter experience, no statistically significant differences in arrhythmia recurrence were observed between PulseSelect pulsed field ablation and fourth-generation cryoballoon ablation at one-year follow-up. However, the study was not designed to establish equivalence or non-inferiority between treatment strategies. Rhythm outcomes remained consistent across different analytical approaches, including varying blanking period definitions, supporting the robustness of the observed results. Both technologies were associated with substantial and clinically meaningful symptom improvement.

## Supplementary Information

Below is the link to the electronic supplementary material.

Supplementary Material 1

## Data Availability

The data underlying this article will be shared by the corresponding author upon reasonable request, subject to approval by the participating centers and in accordance with applicable ethical and privacy regulations.
