# Supplementary material for: Single-shot ablation for AF: Real-world procedural and one-year outcomes with the newly adopted PulseSelect PFA compared with cryoballoon from the 1STOP project
Source: J Interv Card Electrophysiol. 2026 Jul 4;69(9):2831–43. doi: 10.1007/s10840-026-02392-w (PMC13769695; doi:10.1007/s10840-026-02392-w)
Supplement: Supplementary file 1 [file 10840_2026_2392_MOESM1_ESM.docx]

***Supplementary Tables S1A and S1B:*** Multivariable Cox proportional hazards regression analyses for atrial arrhythmia recurrence at 12 months. Model 1 included ablation technology, anesthesia strategy, and AF type. Model 2 included ablation technology, age, and AF type. Separate models were constructed to avoid overfitting given the limited number of recurrence events observed during follow-up.

**Table S1A**

| **Variable** | **Adjusted HR (95% CI)** | **p-value** |
| --- | --- | --- |
| PulseSelect vs Cryoballoon | 2.305 (0.772–6.882) | 0.135 |
| General anesthesia vs other strategies | 0.435 (0.128–1.482) | 0.183 |
| Persistent/Long-standing persistent AF vs Paroxysmal AF | 1.134 (0.321–4.014) | 0.845 |

**Table S1B**

| **Variable** | **Adjusted HR (95% CI)** | **p-value** |
| --- | --- | --- |
| PulseSelect vs Cryoballoon | 1.438 (0.550–3.762) | 0.459 |
| Age (per 1-year increase) | 1.002 (0.953–1.053) | 0.949 |
| Persistent/Long-standing persistent AF vs Paroxysmal AF | 1.375 (0.445–4.252) | 0.580 |
